# The challenges of living with and managing epidermolysis bullosa: insights from patients and caregivers

**DOI:** 10.1186/s13023-019-1279-y

**Published:** 2020-01-03

**Authors:** Anna L. Bruckner, Michael Losow, Jayson Wisk, Nita Patel, Allen Reha, Hjalmar Lagast, Jamie Gault, Jayne Gershkowitz, Brett Kopelan, Michael Hund, Dedee F. Murrell

**Affiliations:** 1 Children’s Hospital Colorado, University of Colorado School of Medicine, 13123 E 16th Ave, B570, Aurora, CO 80045 USA; 2 Amicus Therapeutics, Inc, 1 Cedar Brook Drive, Cranbury, NJ 08512 USA; 3 Debra of America, 75 Broad St #300, New York, NY 10004 USA; 4 EB Research Partnership, 132 East 43rd St, Suite 432, New York, NY 10017 USA; 5 St. George-Hospital, University of Sydney, NSW, 2052 Australia

**Keywords:** Caregivers, Disease burden, Epidermolysis bullosa, Management, Patients, Survey, Quality of life, Financial burden, Wound care

## Abstract

**Background:**

Little information is available regarding the burden of living with and managing epidermolysis bullosa, including the distinct challenges faced by patients with different disease types/subtypes.

**Methods:**

A 90-question/item survey was developed to collect demographics, diagnostic data, management practices, and burden of illness information for patients with epidermolysis bullosa living in the United States. Recruitment was conducted via email and social media in partnership with epidermolysis bullosa patient advocacy organizations in the United States, and the survey was conducted via telephone interview by a third-party health research firm. Respondents aged ≥ 18 years with a confirmed diagnosis of epidermolysis bullosa or caring for a patient with a confirmed diagnosis of epidermolysis bullosa were eligible to participate in the survey.

**Results:**

In total, 156 responses were received from patients (*n* = 63) and caregivers (*n* = 93) representing the epidermolysis bullosa types of simplex, junctional, and dystrophic (subtypes: dominant and recessive). A large proportion of patients (21%) and caregivers (32%) reported that the condition was severe or very severe, and 19% of patients and 26% of caregivers reported a visit to an emergency department in the 12 months prior to the survey. Among the types/subtypes represented, recessive dystrophic epidermolysis bullosa results in the greatest wound burden, with approximately 60% of patients and caregivers reporting wounds covering > 30% of total body area. Wound care is time consuming and commonly requires significant caregiver assistance. Therapeutic options are urgently needed and reducing the number and severity of wounds was generally ranked as the most important treatment factor.

**Conclusions:**

Survey responses demonstrate that epidermolysis bullosa places a considerable burden on patients, their caregivers, and their families. The limitations caused by epidermolysis bullosa mean that both patients and caregivers must make difficult choices and compromises regarding education, career, and home life. Finally, survey results indicate that epidermolysis bullosa negatively impacts quality of life and causes financial burden to patients and their families.

## Background

Epidermolysis bullosa is a rare, often severe genetic disorder characterized by mechanical fragility and blistering or erosions of the skin, mucosa, or epithelial lining of organs in response to minimal trauma [1, 2]. In addition to skin blistering, open wounds, and scarring, severe epidermolysis bullosa can produce extracutaneous manifestations including abnormalities of the gastrointestinal, cardiovascular, and genitourinary systems, as well as the eyes and oral cavities, and is associated with increased risk of premature death [2–4].

Multiple gene mutations affecting proteins responsible for skin integrity have been reported, including keratins (*KRT5*, *KRT14*), laminin-332 (*LAMA3, LAMB3, LAMC2*), dystonin epithelial isoform (*DST*), and collagen types VII and XVII (*COL7A1, COL17A1*) [4, 5]. These mutations result in several disease types and subtypes classified based upon the Stet of the cleavage plane in the skin, each with differing presentation [3, 4]. The four major types of epidermolysis bullosa are epidermolysis bullosa simplex (70%), dystrophic epidermolysis bullosa (25%), junctional epidermolysis bullosa (5%), and Kindler syndrome; however, the first 3 subtypes account for ~ 99% of the patient population [2, 5, 6].

The National Epidermolysis Bullosa Registry estimated that the overall prevalence of epidermolysis bullosa in the United States is 11.1 per one million live births, with an incidence of 1 in every 51,000 live births [7]. Others estimate the incidence of epidermolysis bullosa to be 1 in every 20,000 live births, with approximately 30,000 individuals affected in the United States [8]. Symptoms typically appear around the time of birth, although lesions may not appear in some individuals until adolescence or later, and accurate diagnosis may be delayed until adulthood [4].

Currently, there are no approved treatments for epidermolysis bullosa [9], and management focuses primarily on prevention of blistering, wound care, pain reduction, and early recognition and management of extracutaneous complications [1, 2, 10]. However, little information is available regarding patient and caregiver perspectives on the challenges of managing different types of epidermolysis bullosa and the burden of daily living with the condition [11].

We conducted a survey to understand the current manifestations and impact of epidermolysis bullosa from the patient or caregiver’s perspective and to better understand the difficulties in dealing with various subtypes of the disease among patients residing in the United States.

## Methods

### Survey design

A 90-question/item survey was developed in partnership with epidermolysis bullosa patient advocacy organizations in the United States to collect demographics, diagnostic data, management practices, and burden of illness information on patients with epidermolysis bullosa (Additional file 1). The survey was approved by a central institutional review board (New England Independent Review Board). The questions asked the patient or their caregiver to report on the patient, with the exception of one question that assessed life decisions made by the caregiver (for their own life) based on the patient’s epidermolysis bullosa.

It was planned to recruit 200 patients into the survey. Recruitment was conducted via email and social media in partnership with epidermolysis bullosa patient advocacy organizations (Debra of America and EB Research Partnership) in the United States. The survey was conducted via telephone interview by a third-party health research firm (Engage Health, Eagan, MN). Recruitment and interviewing of patients and caregivers took place between April 11, 2017, and July 24, 2017.

Respondents were eligible to participate in the survey if they met the following criteria: aged ≥ 18 years; confirmed diagnosis of epidermolysis bullosa or caring for a patient with a confirmed diagnosis of epidermolysis bullosa; and provided informed consent. Confirmed diagnosis of epidermolysis bullosa was established based on documentation from the patient’s physician that listed both the name of the patient and the diagnosis of epidermolysis bullosa. Examples of these types of documents included, but were not limited to: physician encounter report/visit summary; physician note for special consideration (e.g., a note stating that a pediatric patient needed to bring scissors to camp for dressing changes); dressing change instructions; epidermolysis bullosa care plan; medical statement for receipt of social services; notification of genetic test results and diagnosis; copy of health record from patient portal; letter to referring physician from epidermolysis clinic; and copy of an email from healthcare provider to a patient confirming diagnosis.

### Analysis

Descriptive statistics, including medians, ranges, and percentages, were used to report the results obtained from respondents. No statistical tests were performed.

## Results

### Patients

In total, 210 people accepted the initial invitation to participate in the survey. Of these, survey responses were received from 156 people in 39 states in the United States (Additional file 2). Fifty-four people, who had initially accepted the invitation to participate, were contacted but did not take part in the survey (*n* = 51, never sent proof of an epidermolysis bullosa diagnosis; *n* = 3, scheduled but did not call in for an interview). Individuals with a diagnosis of epidermolysis bullosa accounted for 63 responses (40.4%), and the remaining 93 responses (59.6%) came from caregivers.

Of the 63 patients who independently completed the survey, the median (range) age was 32 (18–70) years with more females (*n* = 47, 74.6%) than males (*n* = 16, 25.4%). In contrast, the median (range) age of the patients whose caregivers provided responses was 7 (0.2–59) years with more males (*n* = 51, 54.8%) than females (*n* = 42, 45.2%). The composition of epidermolysis bullosa types were similar as reported by patients and caregivers; the overall composition was as follows: simplex (*n* = 55, 35.3%), junctional (*n* = 15, 9.6%), dystrophic (subtypes: *n* = 31, dominant [19.9%] and *n* = 53, recessive [34.0%]), and unknown (*n* = 2, 1.3%) (Table 1). For 119 patients, the first epidermolysis bullosa symptoms were noticed at birth. Of 150 respondents (patients and caregivers) who reported age at epidermolysis bullosa diagnosis, 56 (37.3%) patients were diagnosed with epidermolysis bullosa at birth and 85 (56.7%) more patients were diagnosed before the age of 1 year. 48.3% of patients (28 out of 58) and 40.7% of caregivers (37 out of 91) reported that first symptoms were noticed at birth; in addition, 34.5% of patients (20 out of 58) and 53.8% of caregivers (49 out of 91) reported that the time between first symptoms and diagnosis was 1 day to 1 year.

**Table 1:** Demographics of patients with epidermolysis bullosa as reported by patients and caregivers in the survey

Characteristic	Patient-reported					Caregiver-reported				
	Overall^a^ *N* = 63	Simplex *n* = 21	Junctional *n* = 8	Dominant dystrophic *n* = 14	Recessive dystrophic *n* = 19	Overall^a^ *N* = 93	Simplex *n* = 34	Junctional *n* = 7	Dominant dystrophic *n* = 17	Recessive dystrophic *n* = 34
Sex, *n* (%)										
Male	16 (25.4)	3 (14.3)	4 (50.0)	2 (14.3)	7 (36.8)	51 (54.8)	21 (61.8)	4 (57.1)	11 (64.7)	14 (41.2)
Female	47 (74.6)	18 (85.7)	4 (50.0)	12 (85.7)	12 (63.2)	42 (45.2)	13 (38.2)	3 (42.9)	6 (35.3)	20 (58.8)
Age at survey, years, median (range)	32 (18–70)	37 (18–67)	33 (19–63)	31 (19–60)	30 (18–70)	7 (0.2–59)	5 (0.2–59)	13 (0.3–24)	6 (0.3–45)	7 (0.3–56)
Age at first symptoms, years, median (range)	0.0 (0–5)	0.0 (0–5)	0.0 (0–4)	0.0 (0–5)	0.0 (0–0)	0.0 (0–1)	0.0 (0–1)	0.0 (0–0.1)	0.0 (0–0.5)	0.0 (0–0.1)
Age at diagnosis, years, median (range)	0.0 (0–55)	0.5 (0–55)	0.3 (0–6)	0.0 (0–5)	0.0 (0–30)	0.0 (0–3)	0.1 (0–1)	0.1 (0–1.2)	0.1 (0–3)	0.0 (0–3)
Time between symptoms and diagnosis, years, median (range)	0.0 (0–50)	0.1 (0–50)	0.0 (0–2)	0.0 (0–5)	0.0 (0–30)	0.0 (0–3)	0.0 (0–0.5)	0.0 (0–1.1)	0.0 (0–3)	0.0 (0–3)
Method of diagnosis, *n* (%)^b^										
Skin biopsy	29 (46.0)	11 (52.4)	4 (50.0)	5 (35.7)	8 (41.2)	48 (51.6)	13 (38.2)	6 (85.7)	6 (35.3)	22 (64.7)
Genotyping	4 (6.3)	0	0	2 (14.3)	2 (10.5)	23 (24.7)	10 (29.4)	1 (14.3)	2 (11.8)	10 (29.4)
Physician exam	12 (19.0)	5 (23.8)	1 (12.5)	2 (14.3)	4 (21.1)	15 (16.1)	8 (23.5)	2 (28.6)	2 (11.8)	3 (8.8)
Family history	6 (9.5)	1 (4.8)	0	4 (28.6)	1 (5.3)	11 (11.8)	7 (20.6)	0	2 (11.8)	2 (5.9)
Unknown	24 (38.1)	8 (38.1)	4 (50.0)	5 (35.7)	7 (36.8)	18 (19.4)	6 (17.6)	0	8 (47.1)	4 (11.8)
Other^c^	0	0	0	0	0	8 (8.6)	3 (8.8)	1 (14.3)	1 (5.9)	3 (8.8)

^a^Two patients (1 self-reported and 1 caregiver-reported) had another type or unknown type of epidermolysis bullosa. ^b^Respondents could select more than 1 method. ^c^One of each of the following “other” responses were provided: all skin on hands, legs, and arms was peeled back at birth; amniocentesis prior to birth; biopsy done incorrectly; blood work; electron microscopy; genetic testing; immuno-mapping; inherited; and adopted at 18 months

### Burden of disease

**Fig. 1:**
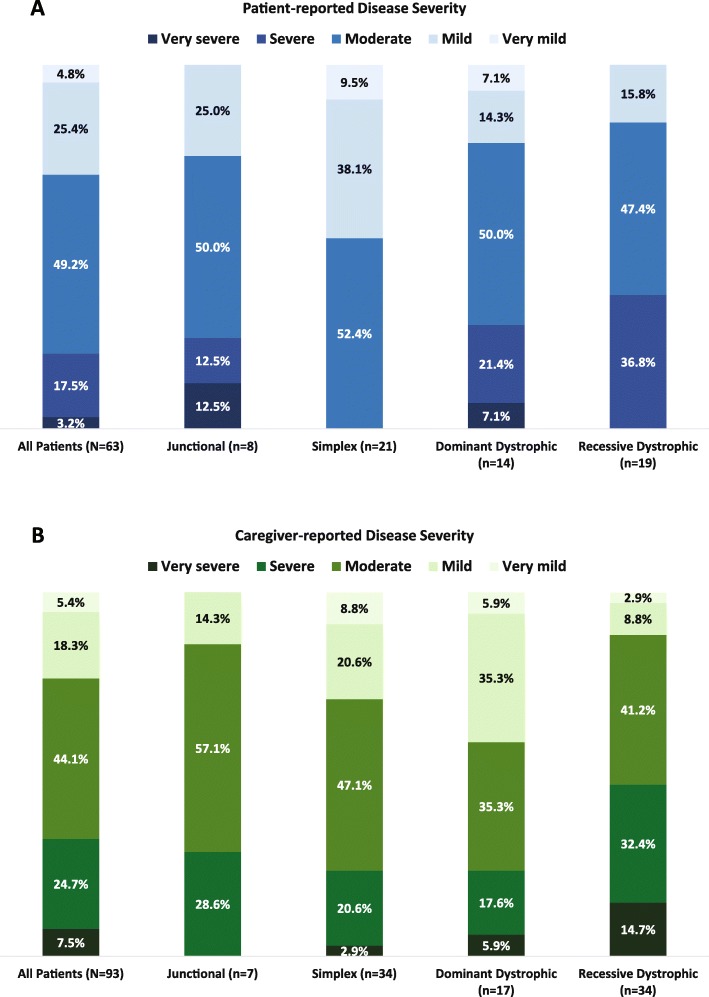
**a** Patient- and **b** caregiver-reported severity of epidermolysis bullosa^a^. ^a^Two patients (1 self-reported and 1 caregiver-reported) had another type or unknown type of epidermolysis bullosa

Almost three-quarters of patients and caregivers rated the epidermolysis bullosa (their own or that of the patient) as moderate to very severe (69.8% of patients [*n* = 44] and 76.3% of caregivers [*n* = 71]) (Fig. 1). On a scale of 1–5 (very mild–very severe), patients with recessive dystrophic epidermolysis bullosa had the highest mean rating of disease severity as reported by both patients and caregivers (3.2 by patients and 3.5 by caregivers), followed by patients with junctional epidermolysis bullosa (3.1 by both patients and caregivers), dominant dystrophic epidermolysis bullosa (3.1 by patients and 2.8 by caregivers), and epidermolysis bullosa simplex (2.4 by patients and 2.9 by caregivers).

**Fig. 2:**
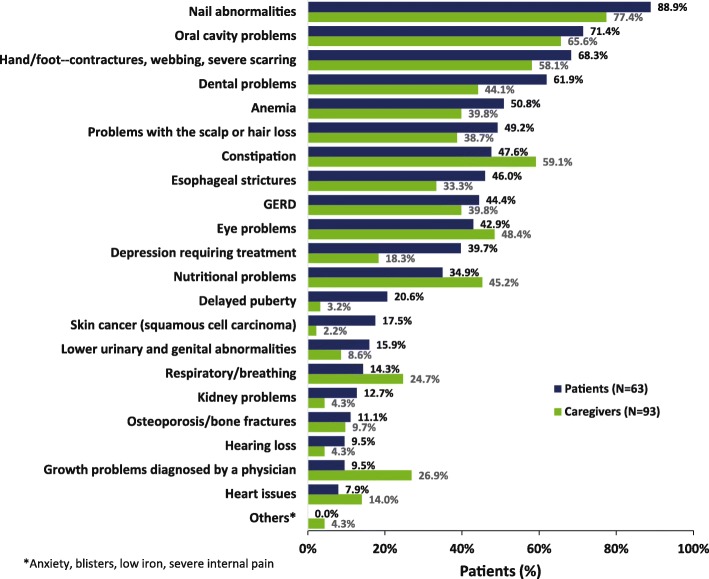
Patient- and caregiver-reported complications experienced due to epidermolysis bullosa. Respondents ticked all applicable answers. ^a^Others: anxiety (*n* = 1); blisters (*n* = 1); low iron (*n* = 1); severe internal pains, cause unknown (*n* = 1). *GERD* gastroesophageal reflux disease

Nearly all respondents (95.2% of patients [*n* = 60] and 95.7% of caregivers [*n* = 89]) reported ≥ 1 complication due to epidermolysis bullosa (Fig. 2). The median numbers of complications reported per individual by patient and caregiver were 7 and 6, respectively. The maximum number of complications reported was 17 (reported by 1 patient and 1 caregiver). Nail abnormalities were common in all types of epidermolysis bullosa. Of note, patients with epidermolysis bullosa simplex had fewer oral cavity and dental problems than patients with other disease types. Conversely, more hand/foot contractures, anemia, esophageal strictures, and nutritional problems were reported in patients with recessive dystrophic epidermolysis bullosa compared with other disease types (Table 2).

**Table 2:** Patient- and caregiver-reported complications experienced due to epidermolysis bullosa by type

Complication, *n* (%)	Patient-reported complications *n* = 62^a^				Caregiver-reported complications *n* = 92^a^			
	Simplex *n* = 21	Junctional *n* = 8	Dominant dystrophic *n* = 14	Recessive dystrophic *n* = 19	Simplex *n* = 34	Junctional *n* = 7	Dominant dystrophic *n* = 17	Recessive dystrophic *n* = 34
Nail abnormalities	15 (71.4)	8 (100)	13 (92.9)	19 (100)	23 (67.6)	5 (71.4)	14 (82.4)	30 (88.2)
Oral cavity problems	7 (33.3)	6 (75.0)	12 (85.7)	19 (100)	12 (35.3)	4 (57.1)	12 (70.6)	32 (94.1)
Hand/foot contractures, webbing, severe scarring	11 (52.4)	6 (75.0)	9 (64.3)	16 (84.2)	15 (44.1)	1 (14.3)	7 (41.2)	30 (88.2)
Constipation	5 (23.8)	5 (62.5)	6 (42.9)	13 (68.4)	15 (44.1)	3 (42.9)	11 (64.7)	25 (73.5)
Dental problems	7 (33.3)	7 (87.5)	8 (57.1)	16 (84.2)	5 (14.7)	6 (85.7)	8 (47.1)	21 (61.8)
Eye problems	8 (38.1)	2 (25.0)	5 (35.7)	11 (57.9)	9 (26.5)	6 (85.7)	4 (23.5)	25 (73.5)
Anemia	4 (19.0)	5 (62.5)	6 (42.9)	16 (84.2)	6 (17.6)	2 (28.6)	4 (23.5)	24 (70.6)
Problems with the scalp or hair loss	5 (23.8)	6 (75.0)	7 (50.0)	13 (68.4)	13 (38.2)	4 (57.1)	2 (11.8)	17 (50.0)
Gastroesophageal reflux disorder	4 (19.0)	5 (62.5)	8 (57.1)	11 (57.9)	7 (20.6)	4 (57.1)	3 (17.6)	22 (64.7)
Nutritional problems	1 (4.8)	3 (37.5)	4 (28.6)	13 (68.4)	8 (23.5)	4 (57.1)	4 (23.5)	25 (73.5)
Esophageal strictures	3 (14.3)	3 (37.5)	4 (28.6)	19 (100)	3 (8.8)	1 (14.3)	3 (17.6)	24 (70.6)
Depression requiring treatment	6 (28.6)	3 (37.5)	9 (64.3)	6 (31.6)	2 (5.9)	3 (42.9)	3 (17.6)	8 (23.5)
Respiratory/breathing complications	2 (9.5)	0	4 (28.6)	2 (10.5)	6 (17.6)	3 (42.9)	4 (23.5)	9 (26.5)
Growth problems diagnosed by a physician	0	1 (12.5)	0	5 (26.3)	8 (23.5)	2 (28.6)	1 (5.9)	13 (38.2)
Heart issues	1 (4.8)	0	2 (14.3)	1 (5.3)	2 (5.9)	0	3 (17.6)	8 (23.5)
Lower urinary and genital abnormalities (eg, urethral stricture, difficulty urinating)	2 (9.5)	2 (25.0)	4 (28.6)	2 (10.5)	2 (5.9)	3 (42.9)	0	2 (5.9)
Delayed puberty	0	5 (62.5)	2 (14.3)	6 (31.6)	0	0	0	3 (8.8)
Osteoporosis/bone fractures	1 (4.8)	2 (25.0)	1 (7.1)	2 (10.5)	1 (2.9)	0	1 (5.9)	7 (20.6)
Skin cancer (squamous cell carcinoma)	4 (19.0)	2 (25.0)	1 (7.1)	3 (15.8)	1 (2.9)	0	1 (5.9)	0
Kidney problems	1 (4.8)	3 (37.5)	3 (21.4)	1 (5.3)	1 (2.9)	0	0	3 (8.8)
Hearing loss	0	1 (12.5)	0	4 (21.1)	1 (2.9)	0	1 (5.9)	1 (2.9)
Other^b^	0	0	0	0	1 (2.9)	0	1 (5.9)	2 (5.9)

^a^Two patients (1 self-reported and 1 caregiver-reported) had another type or unknown type of epidermolysis bullosa and were excluded from analysis. ^b^Other (*n = 1* each): anxiety, blisters, low iron, and several internal pains, cause unknown

In the 12 months prior to the survey, 19.0% of patients (*n* = 12) and 25.8% of caregivers (*n* = 24) had sought care for epidermolysis bullosa from an emergency department, with a mean number of 2 visits. Based on caregivers’ response, patients with recessive dystrophic epidermolysis bullosa were more likely to visit an emergency department (14 out of 34 patients with this epidermolysis bullosa subtype, 41.2%) than patients with other disease subtypes (14.3 to 17.6%). No apparent differences in emergency department visits between disease subtypes were observed based on patients’ responses due to the small number of patient-reported visits to the emergency department.

### Wound burden

About one-third of respondents (31.7% of patients [*n* = 20] and 35.5% of caregivers [*n* = 33]) reported that > 30% of the patient’s total body area was covered by wounds; patients with recessive dystrophic epidermolysis bullosa were more likely to have > 30% of their body covered by wounds (57.9% of patients and 61.8% of caregivers)(Table 3).

**Table 3:** Patient- and caregiver-reported wound burden of epidermolysis bullosa by type/subtype

Wound burden	Patient-reported					Caregiver-reported				
	Overall^a^ *N* = 63	Simplex *n* = 21	Junctional *n* = 8	Dominant dystrophic *n* = 14	Recessive dystrophic *n* = 19	Overall^a^ *N* = 93	Simplex *n* = 34	Junctional *n* = 7	Dominant dystrophic *n* = 17	Recessive dystrophic *n* = 34
Average percentage of body covered by wounds, *n* (%)										
< 10%	22 (34.9)	13 (61.9)	2 (25.0)	5 (35.7)	2 (10.5)	25 (26.9)	12 (35.3)	2 (28.6)	7 (41.2)	4 (11.8)
10–30%	21 (33.3)	6 (28.6)	4 (50.0)	4 (28.6)	6 (31.6)	35 (37.6)	17 (50.0)	3 (42.9)	5 (29.4)	9 (26.5)
> 30%	20 (31.7)	2 (9.5)	2 (25.0)	5 (35.7)	11 (57.9)	33 (35.5)	5 (14.7)	2 (28.6)	5 (29.4)	21 (61.8)
Time required for whole body wound care (including preparation and cleanup), *n* (%)										
< 2 h	45 (71.4)	20 (95.2)	6 (75.0)	11 (78.6)	7 (36.8)	61 (65.6)	30 (88.2)	4 (57.1)	14 (82.4)	12 (35.3)
2–4 h	10 (15.9)	0	2 (25.0)	3 (21.4)	5 (26.3)	23 (24.7)	3 (8.8)	1 (14.3)	3 (17.6)	16 (47.1)
> 4 h	8 (12.7)	1 (4.8)	0	0	7 (36.8)	9 (9.7)	1 (2.9)	2 (28.6)	0	6 (17.6)
Frequency of dressing changes for noninfected wounds, *n* (%)										
Twice daily	2 (3.2)	0	0	1 (7.1)	1 (5.3)	2 (2.2)	2 (5.9)	0	0	0
Every day	25 (39.7)	7 (33.3)	4 (50.0)	7 (50.0)	7 (36.8)	41 (44.1)	13 (38.2)	3 (42.9)	10 (58.8)	15 (44.1)
Every other day	13 (20.6)	1 (4.8)	2 (25.0)	3 (21.4)	7 (36.8)	17 (18.3)	3 (8.8)	1 (14.3)	2 (11.8)	11 (32.4)
Every 3 days	3 (4.8)	0	1 (12.5)	0	2 (10.5)	8 (8.6)	0	2 (28.6)	0	6 (17.6)
As needed	5 (7.9)	4 (19.0)	1 (12.5)	0	0	6 (6.5)	5 (14.7)	0	1 (5.9)	0
Other^b^	15 (23.8)	9 (42.9)	0	3 (21.4)	2 (10.5)	19 (20.4)	11 (32.4)	1 (14.3)	4 (23.5)	2 (5.9)
Frequency of dressing changes for infected wounds, *n* (%)										
2–3x daily	11 (17.5)	6 (28.6)	1 (12.5)	1 (7.1)	3 (15.8)	14 (15.1)	7 (20.6)	2 (28.6)	2 (11.8)	3 (8.8)
Every day	36 (57.1)	9 (42.9)	5 (62.5)	11 (78.6)	10 (52.6)	37 (39.8)	8 (23.5)	2 (28.6)	11 (64.7)	15 (44.1)
Every other day	2 (3.2)	0	0	0	2 (10.5)	5 (5.4)	0	0	0	5 (14.7)
Every 2–3 days	1 (1.6)	0	0	0	1 (5.3)	2 (2.2)	0	1 (14.3)	0	1 (2.9)
As needed	3 (4.8)	3 (14.3)	0	0	0	3 (3.2)	2 (5.9)	0	0	1 (2.9)
Other^c^	5 (7.9)	1 (4.8)	2 (25.0)	1 (7.1)	1 (5.3)	12 (12.9)	6 (17.6)	0	3 (17.6)	3 (8.8)

^a^Two patients (1 self-reported and 1 caregiver-reported) had another type or unknown type of epidermolysis bullosa. ^b^Other (*n* = 1 unless otherwise noted): does not have wounds (*n* = 5); does not dress wounds (*n* = 4); rarely dresses wounds (*n* = 4); does not dress wound unless infected (*n* = 3); try not to dress (*n* = 3); as needed, but prefers to leave open to air; bandage for a day then let air heal; depends on location; depends on wound severity (1–3x per day); depends on wounds; as needed, but if bandaged, every day; less as patient has gotten older; Monday, Wednesday, Friday; most wounds not covered; N/A (not applicable); only rarely when the patient has wounds; only use dressings if oozing; varies; varies (3–4x daily); varies with activities and weather; varies with wound size (2–3x daily). ^c^Other (*n* = 1 unless otherwise noted): rarely (*n* = 4); depends on location (*n* = 2); varies according to needs (*n* = 2); 4–5x daily; depends on how infected; does not wrap; every 3 h; every few hours; Monday, Wednesday, Friday; same as noninfected; several times depending on wound

**Fig. 3:**
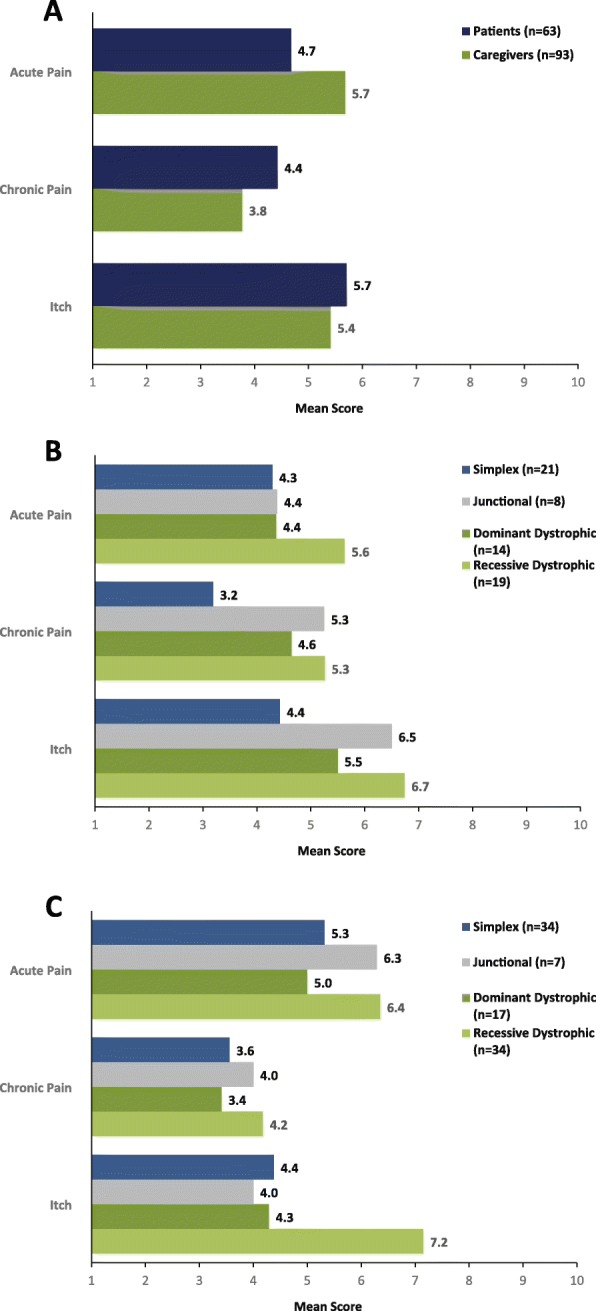
Intensity of wound pain and itch (scored from 1 to 10) in the past 2 weeks as reported by **a** patients and caregivers, **b** as reported by patients by type,^a^ and **c** as reported by caregivers by type^a. a^One patient had another type or unknown type of epidermolysis bullosa and was excluded from analysis

On a scale of 1 (none) to 10 (severe), patients rated their acute pain (mean score 4.7), chronic pain (4.4), and itch (5.7) in the last 2 weeks. Similarly, caregivers rated the patient’s acute pain (mean score 5.7), chronic pain (3.8), and itch (5.4) in the last 2 weeks. Acute pain and itch were reported to be worse in patients with recessive dystrophic disease compared with other types/subtypes (Fig. 3).

Respondents reported that wound care can take several hours (Table 3), with 12.7% of patients and 9.7% of caregivers requiring > 4 h per day to care for wounds. The time required for wound care differed by epidermolysis bullosa type/subtype; patients with recessive dystrophic epidermolysis bullosa reported spending the longest amount of time on wound care whereas caregivers of patients with both junctional and recessive dystrophic subtypes reported spending the most time on wound care.

Patients and caregivers reported changing dressings on infected wounds more often than on noninfected wounds, and the frequency of wound dressing changes differed by epidermolysis bullosa type/subtype (Table 3). Overall, 74.6% of patients (*n* = 47) and 54.8% of caregivers (*n* = 51) stated that they changed the dressings on the same infected wound once or 2 to 3 times per day. In comparison, 42.9% of patients (*n* = 27) and 46.2% of caregivers (*n* = 43) changed the dressings on the same noninfected wound once or twice per day.

Most patients with epidermolysis bullosa required assistance with their wound care regimen. Caregivers reported that of the patients in their care, 66.7% always (*n* = 62), 18.3% sometimes (*n* = 17), and 15.1% never (*n* = 14) required assistance. In contrast, among patients that independently participated in the survey, the frequency of assistance required was always by 12 (19.0%) patients, sometimes by 19 (30.2%) patients, and never by 32 (50.8%) patients. This may partly reflect various patient factors, such as age (i.e., very young children would necessarily require assistance with wound care), physical disability, family preference, etc. According to patients, the most helpful sources of information for learning to care for wounds were a family member (69.8%) or personal experience (trial and error; 39.7%) (respondents could select ≥ 1 source), whereas caregivers benefited almost equally from personal experience (32.3%), patient community (30.1%), family member (26.9%), and epidermolysis bullosa specialists at epidermolysis bullosa treatment centers (25.8%). Most respondents were extremely satisfied (11.1% of patients [*n* = 7] and 17.2% of caregivers [*n* = 16]), satisfied (39.7% of patients [*n* = 25] and 23.7% of caregivers [*n* = 22]), or somewhat satisfied (9.5% of patients [*n* = 6] and 9.7% of caregivers [*n* = 9]) with the wound care guidance received from healthcare specialists.

When respondents were asked to identify the most important factors for a future approved prescription treatment option, the top 5 responses among patients and caregivers were the same: reducing the risk of skin cancer (77.8 and 86.0%), reducing the number and severity of wounds (73.0 and 87.1%), reducing pain (73.0 and 78.5%), accelerating wound healing/closure (71.4 and 80.6%), and reducing risk of infection (69.8 and 76.3%). Reducing itch (57.1 and 74.2%) and decreasing time for dressing change (41.3 and 57.0%) were more important for caregivers than for patients.

### Impact of epidermolysis bullosa on life decisions

#### Patient-reported impact of epidermolysis bullosa on their life decisions

**Fig. 4:**
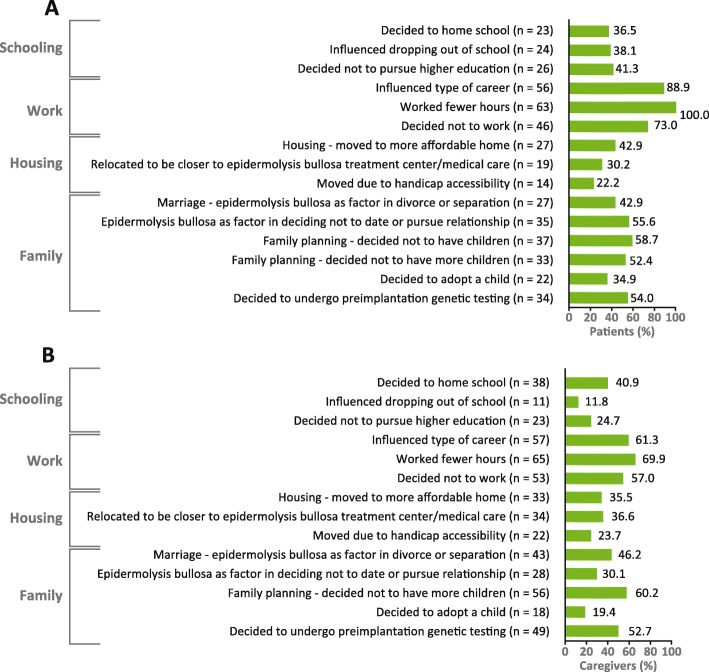
Life decisions resulting from epidermolysis bullosa from the **a** patient’s perspective (*n* = 63) and **b** caregiver’s perspective (*n* = 93). Respondents ticked all applicable answers. Eight of 93 caregivers did not select any of the listed life decisions. Interviewers may or may not have provided the definition of preimplantation genetic testing

The effects of epidermolysis bullosa can be far-reaching, with patients and caregivers making difficult life choices to deal with the impact of the disease. Of 63 patients who answered the question “What life decisions have you made based on epidermolysis bullosa?” all reported working fewer hours because of epidermolysis bullosa; 56 (88.9%) reported that their disease influenced their career choice, and 46 (73.0%) had decided not to work (Fig. 4a).

#### Caregiver-reported impact of epidermolysis bullosa on their life decisions

Of 93 caregivers who answered the question “If you are a parent of a child with epidermolysis bullosa, what life decisions have you made based on your child’s epidermolysis bullosa?” most reported a profound impact on their life choices, with many deciding to reduce their working hours (*n* = 65; 69.9%) or give up work entirely (*n* = 53; 57.0%) (Fig. 4b).

Family planning was an important issue, to reduce the chances of having more children with epidermolysis bullosa. Many parents of children with epidermolysis bullosa reported that the disease was a factor in a divorce or separation (*n* = 43, 46.2%).

### Impact of epidermolysis bullosa on quality of life

Both patients and caregivers reported that epidermolysis bullosa impacted the patient’s quality of life and that a broad range of daily activities were negatively affected. Epidermolysis bullosa clearly interfered with patients’ ability to move around their home, bathe or shower, write, eat, sleep, shop, participate in sports, and play (Table 4). Epidermolysis bullosa also caused physical pain beyond that stemming from wounds, with 39.7% of patients and 48.4% of caregivers reporting occasional pain, 22.2% of patients and 15.1% of caregivers reporting constant pain, and 15.9% of patients and 11.8% of caregivers reporting frequent pain, for themselves and for the patient in their care, respectively. To assess the emotional burden of epidermolysis bullosa on the patient, patients rated for themselves and caregivers rated for the patient in their care, the level of frustration, embarrassment, worry/anxiety, and depression on a scale of 1 to 10 where 1 was defined as “*do not feel*” and 10 was defined as “*feel very strongly.*” A higher level of negative emotions was reported by the patient themselves than what caregivers estimated for the patient. A rating of ≥ 5 was reported by 88.9% of patients and 82.8% of caregivers for frustrated, 74.6% of patients and 52.7% of caregivers for embarrassed, 69.8% of patients and 57.0% of caregivers for worried or anxious, and 66.6% of patients and 34.4% of caregivers for depressed. Finally, epidermolysis bullosa negatively affected socialization of the patient, such as the ability to make new friends, with 44.4% of patients and 32.3% of caregivers reporting “a lot” of or “extensive” impact.

**Table 4:** Patient- and caregiver-reported impact of epidermolysis bullosa on activities of daily living

Does epidermolysis bullosa…	Patient-reported					Caregiver-reported				
	Overall^a^ *N* = 63	Simplex *n* = 21	Junctional *n* = 8	Dominant dystrophic *n* = 14	Recessive dystrophic *n* = 19	Overall^a^ *N* = 93	Simplex *n* = 34	Junctional *n* = 7	Dominant dystrophic *n* = 17	Recessive dystrophic *n* = 34
Affect ability to move around home? *n* (%)										
Severely	5 (7.9)	0	1 (12.5)	2 (14.3)	2 (10.5)	6 (6.5)	0	1 (14.3)	0	5 (14.7)
A lot	12 (19.0)	3 (14.3)	2 (25.0)	4 (28.6)	3 (15.8)	16 (17.2)	6 (17.6)	3 (42.9)	4 (23.5)	3 (8.8)
A Little	32 (50.8)	11 (52.4)	4 (50.0)	6 (42.9)	10 (52.6)	52 (55.9)	21 (61.8)	1 (14.3)	8 (47.1)	21 (61.8)
Not at all	14 (22.2)	7 (33.3)	1 (12.5)	2 (14.3)	4 (21.1)	19 (20.4)	7 (20.6)	2 (28.6)	5 (29.4)	5 (14.7)
Affect ability to bathe or shower? *n* (%)										
Yes, needs help every time	4 (6.3)	0	0	0	4 (21.1)	40 (43.0)	9 (26.5)	4 (57.1)	5 (29.4)	22 (64.7)
Yes, needs help most of the time	4 (6.3)	0	1 (12.5)	2 (14.3)	1 (5.3)	5 (5.4)	1 (2.9)	0	2 (11.8)	2 (5.9)
Yes, sometimes needs help	17 (27.0)	1 (4.8)	2 (25.0)	6 (42.9)	7 (36.8)	18 (19.4)	6 (17.6)	1 (14.3)	4 (23.5)	6 (17.6)
No, no impact	36 (57.1)	19 (90.5)	5 (62.5)	6 (42.9)	6 (31.6)	25 (26.9)	16 (47.1)	2 (28.6)	5 (29.4)	2 (5.9)
N/A	2 (3.2)	1 (4.8)	0	0	1 (5.3)	5 (5.4)	2 (5.9)	0	1 (5.9)	2 (5.9)
Affect ability to write? *n* (%)										
Cannot write due to EB	2 (3.2)	0	0	0	2 (10.5)	2 (2.2)	0	0	1 (5.9)	1 (2.9)
Easier to type	15 (23.8)	4 (19.0)	1 (12.5)	5 (35.7)	4 (21.1)	21 (22.6)	8 (23.5)	2 (28.6)	2 (11.8)	8 (23.5)
Difficult to grip pen	14 (22.2)	4 (19.0)	6 (75.0)	2 (14.3)	2 (10.5)	19 (20.4)	5 (14.7)	0	3 (17.6)	11 (32.4)
Does not interfere	32 (50.8)	13 (61.9)	1 (12.5)	7 (50.0)	11 (57.9)	27 (29.0)	10 (29.4)	4 (57.1)	5 (29.4)	8 (23.5)
N/A	0	0	0	0	0	24 (25.8)	11 (32.4)	1 (14.3)	6 (35.3)	6 (17.6)
Affect ability to eat? *n* (%)										
Always rely on tube	2 (3.2)	0	0	0	2 (10.5)	13 (14.0)	1 (2.9)	2 (28.6)	0	10 (29.4)
Sometimes rely on tube	0	0	0	0	0	5 (5.4)	0	0	1 (5.9)	4 (11.8)
A lot	18 (28.6)	0	3 (37.5)	3 (21.4)	12 (63.2)	16 (17.2)	3 (8.8)	1 (14.3)	3 (17.6)	9 (26.5)
A little	16 (25.4)	5 (23.8)	2 (25.0)	6 (42.9)	2 (10.5)	23 (24.7)	8 (23.5)	2 (28.6)	7 (41.2)	6 (17.6)
No, eat normally	27 (42.9)	16 (76.2)	3 (37.5)	5 (35.7)	3 (15.8)	35 (37.6)	22 (64.7)	2 (28.6)	5 (29.4)	5 (14.7)
N/A	0	0	0	0	0	1 (1.1)	0	0	1 (5.9)	0
Affect ability to sleep? *n* (%)										
Nightly	10 (5.9)	1 (4.8)	2 (25.0)	2 (14.3)	5 (26.3)	15 (16.1)	2 (5.9)	2 (28.6)	1 (5.9)	10 (29.4)
Most nights	9 (14.3)	1 (4.8)	0	3 (21.4)	5 (26.3)	21 (22.6)	8 (23.5)	2 (28.6)	4 (23.5)	6 (17.6)
A little	30 (47.6)	11 (52.4)	4 (50.0)	8 (57.1)	6 (31.6)	30 (32.3)	12 (35.3)	0	3 (17.6)	15 (44.1)
No, sleep normally	14 (22.2)	8 (38.1)	2 (25.0)	1 (7.1)	3 (15.8)	26 (28.0)	12 (35.3)	3 (42.9)	8 (47.1)	3 (8.8)
N/A	0	0	0	0	0	1 (1.1)	0	0	1 (5.9)	0
Affect ability to shop? *n* (%)										
Yes^b^	6 (9.5)	3 (14.3)	0	2 (14.3)	1 (5.3)	4 (4.3)	1 (2.9)	0	1 (5.9)	2 (5.9)
Needs help all the time	6 (9.5)	0	1 (12.5)	1 (7.1)	4 (21.1)	9 (0.7)	2 (5.9)	0	0	7 (20.6)
A lot	6 (9.5)	1 (4.8)	1 (12.5)	1 (7.1)	3 (15.8)	3 (3.2)	2 (5.9)	1 (14.3)	0	0
A little	26 (41.3)	8 (38.1)	4 (50.0)	5 (35.7)	9 (47.4)	17 (18.3)	3 (8.8)	2 (28.6)	5 (29.4)	7 (20.6)
No, not at all	19 (30.2)	9 (42.9)	2 (25.0)	5 (35.7)	2 (10.5)	20 (21.5)	10 (29.4)	2 (28.6)	2 (11.8)	5 (14.7)
N/A	0	0	0	0	0	40 (43.0)	16 (47.1)	2 (28.6)	9 (52.9)	13 (38.2)
Affect ability to play? *n* (%)										
Always	8 (12.7)	0	1 (12.5)	2 (14.3)	5 (26.3)	14 (15.1)	2 (5.9)	3 (42.9)	1 (5.9)	8 (23.5)
A lot	21 (33.3)	6 (28.6)	4 (50.0)	5 (35.7)	6 (31.6)	30 (32.5)	11 (32.4)	1 (14.3)	5 (29.4)	13 (38.2)
A little	18 (28.6)	6 (28.6)	3 (37.5)	3 (21.4)	6 (31.6)	34 (36.6)	11 (32.4)	2 (28.6)	8 (47.1)	12 (35.3)
No	16 (25.4)	9 (42.9)	0	4 (28.6)	2 (10.5)	15 (16.1)	10 (29.4)	1 (14.3)	3 (17.6)	1 (2.9)

*EB* epidermolysis bullosa, *N/A* not applicable

^**a**^Two patients (1 self-reported and 1 caregiver-reported) had another type or unknown type of epidermolysis bullosa. ^b^Yes, (*n = 1* unless otherwise noted): always use a wheelchair when shopping (*n = 3*), does not like to go out, feet get sore with walking and shoes are a problem, feet wounds, heavy things can be a problem on hands, it is hard to get around, scarring on hands makes it hard to grasp things requiring assistance, the need to purchase specific clothing gets tiring

### Financial burden

Survey results indicate that epidermolysis bullosa causes financial burden for a majority of patients and their caregivers. Thirty-two percent (31.7%) of patients and 20.4% of caregivers reported “a lot” of financial burden from epidermolysis bullosa; in addition, 22.2% of patients and 37.6% of caregivers reported “a moderate amount.” Most patients had health care coverage (95.2% of patient respondents and 96.8% of patients whose caregivers responded). Among patient respondents, the most common types of health care coverage were commercial through employer (36.5%) and Medicare (27.0%) or Medicaid (23.8%). Among caregivers, the most common types of health care coverage for the patient were commercial through employer (52.7%), and Medicaid (41.9%). However, not all epidermolysis bullosa-related expenses were reimbursed. The most commonly reported expenses that were not reimbursed by a healthcare plan were over-the-counter medications (81.0 and 77.4% as reported by patients and caregivers, respectively), dressing and wound supplies (65.1 and 63.4%), prescription medications (44.0 and 38.7%), and physician visits (38.1 and 32.3%). The mean amount (USD) of unreimbursed expenses incurred for epidermolysis bullosa care was $262.34 and $682.16 per month as reported by patients and caregivers, respectively.

## Discussion

Epidermolysis bullosa is a rare disease that causes significant morbidity and mortality to affected individuals [1, 4]. Previous reports indicate that epidermolysis bullosa has a significant negative impact on health-related quality of life and places a substantial socioeconomic burden on patients with epidermolysis bullosa and their caregivers [12–16]. The survey reported herein aimed to obtain insight into the impact of epidermolysis bullosa on daily life and activities of patients and their caregivers, and to better understand the different types/subtypes of the condition.

The results of the survey indicate that epidermolysis bullosa places a considerable burden on patients. Many patients and caregivers (21 and 32%) reported that the disease was severe or very severe, and the majority reported at least one additional complication. Furthermore, 19% of patients and 25.8% of caregivers had sought care for epidermolysis bullosa in an emergency department in the 12 months prior to the survey. Some differences were seen between the different types/subtypes of the condition: patients with recessive dystrophic epidermolysis bullosa were more likely to report wounds on > 30% of their body surface and severe/very severe symptoms compared with patients with epidermolysis bullosa simplex (57.9% vs 9.5% and 37% versus 0%, respectively, based on patients’ response). These findings are consistent with observations in the clinical setting, where recessive dystrophic epidermolysis bullosa is regarded as the most severe epidermolysis bullosa type beyond the neonatal/early infancy period.

Wound care was reported to be time consuming and commonly required caregiver assistance. Again, patients with recessive dystrophic epidermolysis bullosa reported the greatest disease burden, spending the longest amount of time on wound care (37% spent > 4 h/day), and recording the highest levels of acute pain (5.6 out of 10) and itch (6.7 out of 10). Most patients with epidermolysis bullosa and caregivers stated that they had learned to care for wounds by trial and error, or from family members and the patient community rather than from healthcare professionals, suggesting that this is an aspect of disease management that requires more attention at the time of initial diagnosis and during subsequent clinic visits. However, of those who did receive wound care guidance from specialists, most were satisfied with the outcome.

It is clear that both patients with epidermolysis bullosa and their caregivers must make difficult choices and compromises regarding education, career, and personal life. Often, the limitations caused by epidermolysis bullosa result in decreased academic and professional achievements, the consequences of which add to the burden of epidermolysis bullosa. Although this survey was not designed to evaluate the full range of psychological effects of the condition, patients with disfiguring skin conditions have reported suffering from poor self-esteem, anxiety, and depression; caregivers report feelings of stress, guilt, and isolation, further impacting overall well-being [4, 14]. Results of our survey support these observations, with patients and caregivers reporting that epidermolysis bullosa negatively impacts activities of daily living, socialization, and emotional well-being. Finally, survey results suggest that the additional expenses incurred by epidermolysis bullosa cause a financial burden for patients, their caregivers, and their families. Our results are in agreement with those from a recent survey of 60 families affected by epidermolysis bullosa in France, which found that parents of children with epidermolysis bullosa experience substantial social, professional, and economic burden [17].

Despite advances in the understanding of the underlying pathophysiology of epidermolysis bullosa, to date, no treatments have been approved by regulatory authorities [3, 9]. Current clinical trials for potential targeted therapies include gene therapy, cell-based therapies, and protein replacement therapy [1, 3]. Patients and caregivers in this survey, and in prior publications [18], have indicated that reducing the number and severity of wounds and decreasing pain are among the main priorities in addition to reducing the risk of skin cancer, suggesting a potential key therapeutic role for topical creams or gels, several of which are also in clinical development [4].

Although surveys can be excellent tools to elicit feedback from patients and identify aspects of the care experience that may need improvement, they also have inherent limitations. One limitation of all surveys is low participation rate, which may suggest a selection bias. In the current study, more than a quarter of initial respondents who expressed interest in participation ultimately failed to complete the survey. Data bias due to question non-responses may also exist. In addition, the reliability of survey data is dependent on the accuracy of the answers provided. Responses provided by caregivers on behalf of patients may be different from those provided by patients themselves. Survey answers could lead to erroneous data because certain options (particularly subjective ratings) may be interpreted differently by respondents; in addition, respondents may not feel comfortable providing honest answers that present themselves in an unfavorable manner. No statistical tests were performed, limiting the comparison of different epidermolysis bullosa types/subtypes.

## Conclusions

These survey results provide valuable information on the commonalities and differences for different types/subtypes of epidermolysis bullosa and confirm that the disease places a substantial burden on patients, their caregivers, and their families. Among the types/subtypes represented, patients and caregivers reported that recessive dystrophic epidermolysis bullosa had the greatest wound burden and acute pain, including the highest rating of disease severity and the highest percentage of body surface area covered by wounds. Wound care is time consuming and commonly requires caregiver assistance. Reducing the number and severity of wounds, pain, and risk of skin cancer were ranked among the most important factors in a future treatment option. The limitations caused by epidermolysis bullosa mean that both patients and caregivers must make difficult choices and compromises regarding schooling, work, housing, relationships, and family planning. Epidermolysis bullosa also negatively impacts quality of life and causes financial burden for patients, their caregivers, and their families.

## Supplementary information

**Additional file 1. **Patient Survey. Copy of patient survey

**Additional file 2.** Patient Enrollment Map. US map showing number of enrolled patients per state.

## Data Availability

The datasets used and/or analyzed during the current study are available from the corresponding author on reasonable request.

## Abbreviations

N/A: Not applicable
USD: United States dollars
