# Supplementary material for: The challenges of living with and managing epidermolysis bullosa: insights from patients and caregivers
Source: Orphanet J Rare Dis. 2020 Jan 3;15:1. doi: 10.1186/s13023-019-1279-y (PMC6942340; doi:10.1186/s13023-019-1279-y)
Supplement: Supplementary file 2 — Additional file 2. Patient Enrollment Map. US map showing number of enrolled patients per state. [file 13023_2019_1279_MOESM2_ESM.pdf]

Amicus US EB Study -- Interviewer Data Collection Form

Page 1

Welcome

This Data Collection Site has been designed as a web based application to collect interview data and to ensure that all questions are asked and collected the same way.

Notes for interviewers;

1. Approximately 15 min before the call, log into this site.
2. Copy the patient information from the invitation that you were sent
3. Hit next to go to the next page.

Engage Health, Inc.

Amicus US EB Study -- Interviewer Data Collection Form

Page 2

Demographic Information

1. Name of Interviewer:*

   [ ]

2. Interviewer, in the invitation to do this interview, it was noted if the "Proof of EB Form" was received. Has this form been received?*

   ○ Yes ○ No

3. Interviewer, in the invitation that you received to do this interview, the patient's type of EB was noted. Please select it here;

   ○ EB Simplex- Localized (Weber-Cockayne)
   ○ EB Simplex- Generalized Severe (Dowling-Meara)
   ○ EB Simplex- Generalized Intermediate (Koebner)
   ○ EB Simplex- I dont know the specific subtype
   ○ Recessive Dystrophic EB- Generalized Severe (Hallopeau-Siemens)
   ○ Recessive Dystrophic EB- Generalized Intermediate (non-Hallopeau-Siemens)
   ○ Recessive Dystrophic EB- I dont know the specific subtype
   ○ Dominant Dystrophic EB- Generalized (Cockayne-Touraine and Pasini)
   ○ Dominant Dystrophic EB-I dont know the specific subtype
   ○ Junctional EB-Generalized Intermediate (Non-Herlitz)
   ○ Junctional EB-Generalized Severe (Herlitz)
   ○ Junctional EB- I dont know the specific subtype
   ○ Kindler
   ○ Other, please specify

   [ ]

4. **Note to Interviewer**

   From the meeting notice that has been sent to you, copy and paste the patient information below. Please be careful when entering as the phone will be important for you to dial correctly, and and the address will be needed to send the compensation check.

   2. Once you have entered this information, and **at the allotted time**, dial the patient at the number provided. If he/she is not there, leave a message

   ", this is ___ from Engage Health calling for our scheduled interview. It sounds like you are running late. I will give you 2 mintues or so and try again". Then hang up and try again in 2 minutes.

   If you try again and they are not there, let them know to contact us to reschedule *", this is ___ again from Engage Health*

calling for our scheduled interview. Since you are not there I will assume this no longer works for you. If you are interested in rescheduling, you can contact *to do so. Thank you"* then hang up.

3. Once they are on the phone;

", this is ___ from Engage Health calling for our interview, I appreciate you participating today. We will be together about an hour for this interview. I want to remind you that if there is a question tha tis too uncomfortable for you to answer, just let me know. At certain points I may move the interview along...please forgive me in advance-- I dont mean to be rude but there are quite a few questions to get through and I want to make sure we get to it all. OK? Before we get into the discussion at hand, I want to check a few details.... I have that you are at ADDRESS is that right?" (if not, correct it). Once filled in and varified, go to the next question.

First Name:	
Last Name:	
Street Address:	
City:	
State (i.e. OH):	
Zip Code:	
Country:	
Email Address:	
Telephone (with area code, no other characters i.e. XXXXXXXXXX):	

5. *When you signed up to do this market research, you noted that you are is that correct?*

If they are the parent:
*As we go through the interview, the questions are sometimes worded "you" or "your". In all cases, we are actually asking about your son/daugher. OK?"**

- ○ Individual with Epidermolysis Bullosa (age 18 or older)
- ○ Parent/Guardian/Caretaker of individual with Epidermolysis Bullosa

6. *When you signed up to do this market research, you noted that you had INSERT NAME OF EB SUBTYPE HERE. Is that correct?*

Note to interviewer, if this is not correct, click here to provide the correct type. There has been a change in how EB is described. The patient may know the older name, which is found in the parentheses.

*

- ○ EB Simplex- Localized (Weber-Cockayne)
- ○ EB Simplex- Generalized Severe (Dowling-Meara)
- ○ EB Simplex- Generalized Intermediate (Koebner)
- ○ EB Simplex- I dont know the specific subtype
- ○ Recessive Dystrophic EB- Generalized Severe (Hallopeau-Siemens)
- ○ Recessive Dystrophic EB- Generalized Intermediate (non-Hallopeau-Siemens)
- ○ Recessive Dystrophic EB- I dont know the specific subtype
- ○ Dominant Dystrophic EB- Generalized (Cockayne-Touraine and Pasini)
- ○ Dominant Dystrophic EB-I dont know the specific subtype

- ○ Junctional EB-Generalized Intermediate (Non-Herlitz)
- ○ Junctional EB-Generalized Severe (Herlitz)
- ○ Junctional EB- I dont know the specific subtype
- ○ Kindler
- ○ Other, please specify

7. What is the gender of the person who is the subject of the survey:*
   - ○ Male
   - ○ Female

8. *What is the year of birth (**XXXX**) and current age (**XX**) of the person who is the subject of the survey.*

   If they decline to answer, put "99". Put all monthly increments in decimals, so if they say "one year, 6 months", put 1.5*

	Year	Age
Year of Birth / Age		

9. *Please provide your place of residence:**

   If they choose not to answer write "No Answer". Please use Proper Case with the first letter capitalized and the rest not capitalized. For state, use the two letter abbreviation (i.e. MN) and if United States, put "United States"

	City	State/Province	Country
Place of Residence:			

Amicus US EB Study -- Interviewer Data Collection Form

Page 3

Individual with EB Information

10. *Please note the age (of the patient) at which you noticed the first symptoms of EB**

(Please specify days, weeks, months, or years after your numerical value (i.e. 10 years or 16 weeks)) Interviewer: be specific as possible using decimal places; if they say "2 years, 6 months" note "2.5 years"

Age: ______

11. *At what age (of the patient) was diagnosis of EB made?* *

(Please specify days, weeks, months, or years after your numerical value (i.e. 10 years or 16 weeks)) Interviewer: be specific as possible using decimal places; if they say "2 years, 6 months" note "2.5 years"

Age: ______

12. *How was the diagnosis confirmed, please tell me ALL of the following that apply:* *

(Please check all that apply)

- ☐ Skin Biopsy
- ☐ Genotyping
- ☐ Physician exam
- ☐ Don't know
- ☐ Other, please specify ______

13. *Which of the following physicians did you/ your child see prior to diagnosis?*

Please select all that apply*

- ☐ Neonatologist
- ☐ Pediatric dermatologist
- ☐ Adult dermatologist
- ☐ Dermatologist (general)
- ☐ Geneticist
- ☐ Don't know
- ☐ None
- ☐ Other, please specify ______

14. *What type of physician made the final diagnosis?*

Please select the option that best applies*

- ☐ Neonatologist
- ☐ Pediatric dermatologist
- ☐ Adult dermatologist
- ☐ Dermatologist (general)
- ☐ Geneticist
- ☐ Pediatrician
- ☐ Don't know
- ☐ Other, please specify

15. *How many of your immediate or extended family members have also been diagnosed with EB?*

Note to interviewer: Fill in each box. For example, if they say "none" put a "0" in the box. If they do not know, put "99" in the box.*

Mother:

Father:

Sister(s):

Brother(s):

Child(ren):

Aunt(s):

Uncle(s):

Niece(s):

Nephew(s):

None:

16. *Do you participate in an EB patient registry? If yes, please give the name of the registry and the approximate date enrolled.*

Note to interviewer: If they dont know the name, put in what they DO know, i.e. "the one of out University of WI". If they dont know the date or are unsure, put "approximately Sept 2014" or whatever they say*

- ○ No
- ○ Not Sure
- ○ Yes, please provide the name of registry

17. *If you answered "no" that you do not participate in an EB registry, please tell me which option best fits the reason why:*

- ○ Never been asked to participate in a patient registry
- ○ No time to be involved in a patient registry
- ○ Not interested in being involved in a patient registry
- ○ Dont know waht a registry is
- ○ Other, please specify

18. *How would you rate the overall severity of you or your child's EB?**

- ○ Very mild
- ○ Mild

- ○ Moderate
- ○ Severe
- ○ Very severe

19. *What complications have you or your child experienced due to your EB?**

(Please check all that apply)

- ☐ Problems with the scalp or hair loss
- ☐ Eye problems
- ☐ Hearing loss
- ☐ Respiratory/breathing
- ☐ Heart issues (e.g. cardiomyopathy- diseases of the heart muscle)
- ☐ Dental problems
- ☐ Oral cavity problems (mouth, lips, teeth, cheeks, tongue and throat)
- ☐ Esophageal strictures (e.g. narrowing of the esophagus)
- ☐ GERD (gastroesophageal reflux disease or "heartburn")
- ☐ Constipation
- ☐ Kidney problems (e.g. renal failure)
- ☐ Lower urinary and genital abnormalities (e.g. urethral stricture, difficulty urinating)
- ☐ Anemia
- ☐ Nutritional problems
- ☐ Growth problems diagnosed by a physician
- ☐ Delayed puberty
- ☐ Osteoporosis (thinning of bones)/ bone fractures
- ☐ Nail abnormalities
- ☐ Hand/foot- contractures, webbing, severe scarring
- ☐ Skin cancer (squamous cell carcinoma)
- ☐ Depression requiring treatment
- ☐ Others, please list all

20. *Have you or your child been diagnosed with any other medical condition?**

- ○ No
- ○ Yes, it (or they) is/are specified here:

Amicus US EB Study -- Interviewer Data Collection Form

Site of Care/Healthcare Professionals

21. *Over the **past year**, who did you routinely see for the **management of your/your child's EB**, how often and in what setting?**

(Please note the number of visits and note "Yes" or "No" to describe if the doctor was hospital based)

	# of visits per year	Hospital Based? (interviewer-note "yes" or "no"
Primary care physician:		
Pediatrician:		
Dermatologist:		
Pediatric dermatologist:		
Other:		

22. *If you noted "other" above, please specify the type of physician here*

23. *If the physician you saw for the routine managment of EB is hospital based, please note the hospital name(s) and the average distance from home, noting if that measurement is in miles or km.*

(Please note average distance from home in either miles or km, just be sure to note which unit of measurement you are using, i.e. 16 miles)

Hospital name(s):

Average distance from home:

24. *Did these visits include multi-disciplinary (EB clinic) care (a team of doctors & other healthcare professionals)?*

○ No
○ Yes, the number of times per year that multi-disciplinary care was included is noted here:

25. *Over the past year, from which office/site did you/your child seek care for EB and how often?**

(Please select "Yes" or "No" from each drop down menu and also note the number of visits)

	Seek Care?	Number of Visits

Emergency room:	-- Please Sel	
Hospital in-patient care:	-- Please Sel	
General Surgeon:	-- Please Sel	
Hand Surgeon:	-- Please Sel	
Ear Nose and Throat Specialist:	-- Please Sel	
Hematologist (Blood Specialist):	-- Please Sel	
Pain Specialist:	-- Please Sel	
Neurologist:	-- Please Sel	
Gastroenterologist:	-- Please Sel	
Cardiologist:	-- Please Sel	
Kidney Doctor:	-- Please Sel	
Oncologist/dermatology-oncologist:	-- Please Sel	
Psychiatrist:	-- Please Sel	
Dentist:	-- Please Sel	

26. *Do you or your child receive care from a health care provider other than a doctor?**

- ○ Often
- ○ Sometimes
- ○ Never

27. *If sometimes or often, what type of assistance is received?*

Check all that apply:

- ☐ Wound care assistance
- ☐ Nutritional support
- ☐ Evaluation of needs
- ☐ Other, please specify

28. *By what professionals?**

(Please select "Yes" or "No" from each drop down menu, also note the number of times per year and location)

	**Seek Care?**	**Number of Times Per Year**	**Location - Interviewer, please note "Homecare" or "At a Center"**
Registered Nurse:	-- Please Se		
Physical Therapist:	-- Please Se		
Social Worker:	-- Please Se		
Occupational Therapist:	-- Please Se		
Psychologist/Therapist:	-- Please Se		
Podiatrist:	-- Please Se		

Chiropractor:	-- Please Se		
Nutritionist:	-- Please Se		
Physical Therapist/ Occupational Therapist:	-- Please Se		
Other:	-- Please Se		

Amicus US EB Study -- Interviewer Data Collection Form

Page 5

Wound Management

29. *The majority of the time, what is the average percent of you or your child's body covered by wounds? Please refer to the image below to correctly estimate the percentage.*

(Definition of Perineum: The area between the anus and the scrotum or vulva)

*

	Greater than 30%	10-30%	Less than 10%
Head (including neck):	○	○	○
Trunk:	○	○	○
Arms:	○	○	○
Legs:	○	○	○
What is the average percent of your/your child's total body that is covered by wounds?:	○	○	○

30. *Are there any wounds whose locations change?*

Please select the option that best describes your experience

○ ALL wounds change locations frequently
○ SOME wounds change locations frequently
○ ALL wounds change locations from time to time
○ SOME wounds change locations from time to time
○ NO wounds change location
○ SOME wounds do not change location

31. *Do you follow a specific wound care regimen for you/your child?**

○ Yes
○ No

32. *If yes, how satisfied are you / your child with it?**

○ Extremely satisfied
○ Satisfied

- ○ Somewhat satisfied
- ○ Neutral
- ○ Somewhat dissatisfied
- ○ Dissatisfied
- ○ Extremely dissatisfied

33. *For the last two weeks, how would you rank the intensity of pain and itch of your/ your child's wounds?*
Please use a scale of 0 to 10 with 0 being "none" and 10 being "severe"

	(0 = None)										(10 = Severe)*
	0	1	2	3	4	5	6	7	8	9	10
Acute pain (Sudden onset--e.g. dressing changes, going to the bathroom, trauma):;;											
Chronic pain (ongoing pain during the day/night):;;											
Itch:;;											

34. *If you noted there was chronic pain, how long did it last?*

Note to interviewer, please note the answer in number of hours. So if they say "a day and a half", put "36 hours" . If they do not know, put "99", if there was none, put "0"*

35. *If you noted there was itch, how long did it last?*

Note to interviewer, please note the answer in number of hours. So if they say "a day and a half", put "36 hours" . If they do not know, put "99", if there was none, put "0"*

36. *Over the past year, how often did you need to use antibiotics/antimicrobials needed to treat infected wounds?*

*

(Please select all that apply)

	Never	Sometimes	Often
Over the counter topical antibiotic/antimicrobial (e.g. bacitracin):	☐	☐	☐
Prescription topical antibotic/antimicrobial:	☐	☐	☐
Oral antibiotic:	☐	☐	☐
Antimicrobial dressing (e.g. silver dressing):	☐	☐	☐
Intravenous / IV antibiotic:	☐	☐	☐

Other:	☐	☐	☐

37. *If you noted "other" above, please describe it here, including the route of administration (i.e. oral, topical)*

[ ]

Engage Health, Inc.

Amicus US EB Study -- Interviewer Data Collection Form

Wound Management

38. *Do you / your child take pain medication prior to bathing and dressing changes?**

○ Always
○ Sometimes
○ Never

39. *If "sometimes" or "always", what types of treatment do you use?**

(Please check all that apply)

☐ Over-the-counter pain medication (eg. Tylenol/acetaminophen, Advil/ibuprofen)
☐ Prescription pain medication i.e.. opioids (e.g. oxycodone, Vicodin, codeine, morphine...)
☐ Other, please specify

40. *Do you or your child use pain medication for chronic pain (on-going pain during the day/night)?**

○ Daily
○ A few times per week
○ Never

41. *If "a few times per week" or "daily", what types of treatment do you use?**

(Please select all that apply)

☐ Over-the-counter pain medication (eg. Tylenol/acetaminophen, Advil/ibuprofen)
☐ Opioids (e.g. oxycodone, Vicodin, codeine, morphine..)
☐ Gabapentin
☐ Cannabinoids
☐ Tricyclic Antidepressants (e.g. amitriptyline, doxepin)
☐ Other, please specify

42. *How often do you/ your child use medication to control itch?**

○ Daily
○ A few times per week
○ As needed
○ Never

43. *If "a few times per week" or "daily", what type of treatment do you use?**

☐ Anti-histamines oral (e.g. Cetirizine, diphenhydramine)

- ☐ Anti-histamine cream/lotion
- ☐ Tricyclic anti-depressant (eg. amitriptyline, doxepin)
- ☐ Ondansetron
- ☐ Topical steroid creams
- ☐ Non-medicated creams/ lotions
- ☐ Other, please specify

Engage Health, Inc.

Amicus US EB Study -- Interviewer Data Collection Form

Page 7

Wound Management

44. *How often do you carry out dressing changes for the same NON-INFECTED wounds?**

- ○ Every day
- ○ Every other day
- ○ Every two days
- ○ Every three days
- ○ Other, please specify

45. *How often do you carry out dressing changes for the same INFECTED wounds?**

- ○ Every day
- ○ Every other day
- ○ Every two days
- ○ Every three days
- ○ Other, please specify

46. *What influences the frequency of dressing changes on a scale of 1-10 with 10 being the most important?*

(1 = Least important 10 = Most important)*

	0	1	2	3	4	5	6	7	8	9	10
Cost of dressings:;;											
Insurance coverage for dressings:;;											
Availability/ convenience:;;											
Effectiveness:;;											
Comfort or minimizing pain/discomfort:;;											

Ease of application / use:;;

Reducing risk of infection:;;

Minimizing wound bleeding/ damage:;;

Doctor's advice/ instructions:;;

Doctor's prescription:;;

Other:;;

47. *If you noted "other" above, please specify it here:*

48. *How long does it take you to carry out the wound care regimen (bathing, changing, dressings...) for the whole body, including preparation & clean up time?**

For the purposes of this question please consider the time to prepare supplies, remove old dressings, bathe or clean the wound, apply therapeutic agent(s), replace dressings and discard used materials:

- ○ Less than 1 Hour
- ○ 1 Hour to 1 Hour 59 minutes
- ○ 2 Hours to 2 Hours 59 minutes
- ○ 3 Hours to 3 Hours 59 minutes
- ○ 4 Hours to 4 Hours 59 minutes
- ○ 5 hours or greater

49. *Do you or your child require assistance with your wound care regimen?**

- ○ Always
- ○ Sometimes
- ○ Never

50. *If "always" or "sometimes" I/my child require(s) help for:*

- ○ The fully body
- ○ Only certain parts of the body

51. *Who helps with the wound care regimen?*

(Please select all that apply)

- ☐ Parent(s)
- ☐ Husband/wife/significant other
- ☐ Other relative(s)
- ☐ Personal care aide
- ☐ Nurse

☐ Friend
☐ I don't have help, but need it
☐ I don't need help

52. *What percentage of time is wound care performed by a family member?* *

○ Never
○ 1-24% of the time
○ 25-49% of the time
○ 50-74% of the time
○ 75-99% of the time
○ Always

53. *What type of dressings do you or your child use most often for wounds?**

(Dressing layers do not include ointment)

	**Product Name**
First or primary layer (directly on wound):	
Secondary layer:	
Outer layer:	
Additional layer: (please note product name AND specify the layer)	

54. *Do you apply ointment or cream to wound before applying a dressing?*

○ No
○ Yes, provide name of cream(s)/ointment(s):

55. *What influences the type of dressing used on a scale of 1-10 with 10 being the most important?*

	**(1 = Least important**									**10 = Most important)***
	**0**	**1**	**2**	**3**	**4**	**5**	**6**	**7**	**8**	**9**	**10**
Cost of dressings:;;											
Insurance coverage for dressings:;;											
Availability/ convenience:;;											
Effectiveness:;;											
Comfort or minimizing pain/discomfort:;;											

Ease of application / use:;;

Reducing risk of infection:;;

Minimizing wound bleeding/ damage:;;

Doctor's advice/ instructions:;;

Doctor's prescription:;;

Other:;;

56. *If **cost** or **insurance coverage** is an important criteria, what would you do differently if you could choose freely?*

(Please select all that apply)

☐ Choose other products
☐ Increase the frequency of dressing changes
☐ Not change anything
☐ Other, please specify

Amicus US EB Study -- Interviewer Data Collection Form

Page 8

Wound Management

57. *How satisfied are you with the* ***guidance/instruction*** *that you receive for wound care from health care specialists?* *

- ○ Extremely satisfied
- ○ Satisfied
- ○ Somewhat satisfied
- ○ Neutral
- ○ Somewhat dissatisfied
- ○ Dissatisfied
- ○ Extremely dissatisfied

58. *Do you/ your child have a written/prescribed wound care plan?* *

- ○ Yes
- ○ Yes, we have a wound care plan but it is not followed
- ○ No

59. *If yes, who made the plan?* *

- ○ Office-based dermatologist
- ○ EB specialist at a hospital-based EB center
- ○ Nurse at hospital-based EB center
- ○ Homecare nurse
- ○ Office-based pediatrician
- ○ Office based primary care physician
- ○ Other, please specify ________

60. *What or who was the most helpful to you when learning how to care for your/ your child's wounds?* *

(Please select all that apply)

- ☐ Office-based dermatologist
- ☐ EB specialist at a hospital-based EB center
- ☐ Nurse at a hospital-based EB center
- ☐ Homecare nurse
- ☐ Office-based pediatrician
- ☐ Primary care physician
- ☐ Patient association
- ☐ Family member
- ☐ Patient community (e.g., patients, relatives of patients)

☐ Social media, (e.g. Facebook)
☐ Personal experience (trial & error)
☐ Other, please specify

61. *Who is the primary prescriber of you /your child's wound care treatments?**

○ Office-based dermatologist
○ EB specialist at a hospital-based EB center
○ Office-based pediatrician
○ Primary care physician / general practitioner
○ Nurse practitioner/physician assistant at a hospital based EB Center
○ None
○ Other, please specify

Engage Health, Inc.

Amicus US EB Study -- Interviewer Data Collection Form

Page 9

Quality of Life Questions

62. *Does EB affect your/your child's ability to move around at home?**

- ○ Not at all
- ○ A little
- ○ A lot
- ○ Severely

63. *Does EB affect your/your child's ability to bathe or shower?**

- ○ No, no impact
- ○ Yes, sometimes need assistance
- ○ Yes, need assistance most of the time
- ○ Yes, need assistance every time bathe/shower
- ○ N/A

64. *Does EB cause physical pain (pain experienced other than pain from wounds)?**

- ○ No pain
- ○ Occasional pain
- ○ Frequent pain
- ○ Constant pain

65. *How does EB affect your/your child's ability to write?**

- ○ It does not interfere with writing
- ○ Difficult to grip a pen
- ○ Easier to type than write
- ○ Cannot write due to EB
- ○ N/A

66. *Does EB affect your/your child's ability to eat?**

- ○ No, eats normally
- ○ A little
- ○ A lot
- ○ Sometimes rely on gastrostomy tube
- ○ Always rely on gastostomy tube
- ○ N/A

67. *Does EB affect your/your child's ability to sleep?**

- ○ No, sleeps normally
- ○ A little
- ○ A lot (most nights)
- ○ Nightly (trouble sleeping every night)
- ○ N/A

68. *Does EB affect your/your child's ability to shop?**
    - ○ No, not at all
    - ○ A little
    - ○ A lot
    - ○ Need assistance all the time
    - ○ N/A
    - ○ Yes, please explain

69. *How does EB affect your/your child's involvement in sports?**
    - ○ No impact
    - ○ Need to be cautious in sports
    - ○ Need to avoid some sports
    - ○ Need to avoid all sports
    - ○ It does not impact sports

70. *Does EB affect you or your child's ability to play?**
    - ○ No
    - ○ A little
    - ○ A lot
    - ○ Always

71. *Has EB caused you/ your child to feel any of the following?*

	(0 = Do not feel							10= Feel very strongly)*			
	**0**	**1**	**2**	**3**	**4**	**5**	**6**	**7**	**8**	**9**	**10**
Frustrated:											
Embarrassed:											
Worried or anxious:											
Depressed:											

72. *How much does EB affect socialization? (ability to make new friends, have friends speak in public)**
    - ○ None, not at all
    - ○ A little
    - ○ A lot

○ Extensive

73. *If yes to the question above, please explain why:*

74. *Have you needed to, or do you need to modify your home (installing ramps, etc...) due to EB?**

○ No, not at all
○ A little
○ A lot
○ Extensive

75. *If yes to question above, list modifications made or needed:*

Engage Health, Inc.

Amicus US EB Study -- Interviewer Data Collection Form

Page 10

Impact of EB on Work/ School

76. *Do you/ your child miss days from work or school due to EB each month?*

Note to interviewer: please note a number and if it is hours, days, weeks per month

○ No
○ Yes, the number of days and the unit of measure is noted here:

[ ]

77. *If you answered "yes" to the question above, what are teh primary reasons?* *

Check all that apply:

☐ Skin complications (e.g. infection, pain)
☐ Non-Skin complications (e.g. stomach problems)
☐ Too exhausted
☐ Not feeling well enough
☐ Medical appointments
☐ Other medical reasons
☐ Financial (e.g. remain eligible for disability compensation)
☐ Other, please specify

[ ]

Engage Health, Inc.

Amicus US EB Study -- Interviewer Data Collection Form

Future Treatment

78. *If there was an approved prescription treatment option, what would be important to you/your child? List the importance on a scale of 1 to 10 with 10 being the most important.*

	(1 = Least important									10 = Most important)*
	0	1	2	3	4	5	6	7	8	9	10
Reduction in the number and severity of wounds:											
Accelerate wound healing / Close existing wounds:											
Decrease time for dressing change:											
Reduce itch:											
Reduce pain:											
Reduce risk of infection:											
Reduce risk of skin cancer (squamous cell carcinoma):											
Other:											

79. *If you noted "other" in the question above, please specify it here:*

Engage Health, Inc.

Amicus US EB Study -- Interviewer Data Collection Form

Cost and Insurance Information

80. *Do you have healthcare Coverage?**

○ Yes
○ No

81. *If yes, what type of insurance do you have?**

(Please check all that apply)

☐ Commercial - provided by employer (eg. Aetna, United Healthcare, Blue Cross/Blue Shield, Kaiser Permanente)
☐ Commercial - private pay
☐ Medicaid
☐ Medicare
☐ Healthcare exchange (eg. Affordable Care Act or "Obamacare")
☐ Government (eg. Tricare, VA, DoD)
☐ Additional/supplemental insurance (e.g. PSI, debra of America, NORD)
☐ Other, please specify

82. *Do you/ your child qualify for disability compensation/assistance?**

○ Yes
○ No

83. *If yes, do you limit your assets to remain eligible for disability compensation/assistance?**

○ Yes
○ No

84. *What expenses are covered by your/ your child's healthcare plan / insurance / government payer?**

(Please check all that apply)

☐ Medications
☐ Physician visits
☐ Dressings and supplies
☐ Physical therapy
☐ Occupational therapy
☐ Dental health
☐ Feeding supplies
☐ Medical equipment (e.g. tubing, pump, wheelchair, walker, others)
☐ Transportation to medical visits

- ☐ Nurse home care
- ☐ Nurse hospital based or doctor's office care
- ☐ Personal care aid
- ☐ ER visits and hospitalizations
- ☐ Home modifications
- ☐ Other, please specify

85. *If you said that dressings and supplies are covered, please note the percent coverage below:*

86. How much financial burden is attributed to EB?*

- ○ None
- ○ A little
- ○ A moderate amount
- ○ A lot

87. *What expenses do you incur for your/ your child's EB care (please consider expenses that are not reimbursed by your healthcare plan, insurance, government payer). Please note if you have this expense, and the approximate cost, per month in dollars.*

Note to Interviewer: they will think of hospitalizatoins per year, so that is the only one to collect by year
*

	**Incur expense**	**$ spent per month (in US dollars)**
Personal care aid:	-- Please Sel	
ER visits & hospitalizations- PER YEAR:	-- Please Sel	
Dental health:	-- Please Sel	
Feeding supplies:	-- Please Sel	
Home modifications:	-- Please Sel	
Medical equipment (tubing, pump, wheelchair, walker, etc.):	-- Please Sel	
Other:	-- Please Sel	
Over the counter medications:	-- Please Sel	
Prescription medications:	-- Please Sel	
Physician visits:	-- Please Sel	
Other healthcare professional visits (e.g. physical therapist, occupational therapist, etc.):	-- Please Sel	
Dressings & supplies:	-- Please Sel	
Transportation to medical visits:	-- Please Sel	
Nurse home care:	-- Please Sel	
Nurse hospital-based or doctor's office care:	-- Please Sel	

88. *If you noted "other" above, please specify it here:*

89. *What life decisions have you/your child made based on EB? Check all that apply:*

Note to interviewer: answer this from the perspective of the patient*

	Yes	No
Influenced type of career:	☐	☐
Worked fewer hours:	☐	☐
Decided not to work:	☐	☐
Decided not to pursue higher education:	☐	☐
Influenced dropping out of school:	☐	☐
Decided to home school:	☐	☐
Marriage- EB a factor in divorce or separation:	☐	☐
EB a factor in deciding not to date or pursue relationship:	☐	☐
Housing- move to more affordable home:	☐	☐
Relocated to be closer to EB treatment center or medical care:	☐	☐
Moved due to handicap accessibility:	☐	☐
Family planning- decided not to have children:	☐	☐
Family planning- decided not to have MORE children:	☐	☐
Decided to adopt a child:	☐	☐
Decided to undergo pre-implantation genetic testing:	☐	☐

90. *If you are a PARENT of a child with EB, what life decisions have youmade based on your child's EB? Check all that apply:*

Note to interviewer: answer this from the perspective of the PARENT*

Yes	No
☐	☐

Decided to undergo pre-implantation genetic testing:		
Influenced type of career:	☐	☐
Worked fewer hours:	☐	☐
Decided not to work:	☐	☐
Decided not to pursue higher education:	☐	☐
Influenced dropping out of school:	☐	☐
Decided to home school:	☐	☐
Marriage- EB a factor in divorce or separation:	☐	☐
EB a factor in deciding not to date or pursue relationship:	☐	☐
Housing- move to more affordable home:	☐	☐
Relocated to be closer to EB treatment center or medical care:	☐	☐
Moved due to handicap accessibility:	☐	☐
Family planning- decided not to have children:	☐	☐
Family planning- decided not to have MORE children:	☐	☐
Decided to adopt a child:	☐	☐

Engage Health, Inc.

Amicus US EB Study -- Interviewer Data Collection Form

Page 13

91. *We are at the end of the questions. Thank you so much for your time and for sharing your experience! As soon as we are done I will let our office know, and they will process your honoraria check. If for some reason you do not recieve it within 10 business days, please let us know.*

    Thanks again, I wish you (your family) all the best!

    Note to interviewer: put here the number of minutes the interview took. Hit DONE on the next page to capture data so that the patient gets paid.

Engage Health, Inc.

Amicus US EB Study -- Interviewer Data Collection Form

Page 14

Thank You

Thank you for taking the survey, we will send your honorarium check shortly.
